# Macular perfusion analysed by optical coherence tomography angiography after uncomplicated phacoemulsification: benefits beyond restoring vision

**DOI:** 10.1186/s12886-021-01837-2

**Published:** 2021-02-05

**Authors:** Ana Križanović, Mirjana Bjeloš, Mladen Bušić, Biljana Kuzmanović Elabjer, Benedict Rak, Nenad Vukojević

**Affiliations:** 1 Department of Ophthalmology, Reference Centre of the Ministry of Health of the Republic of Croatia for Paediatric Ophthalmology and Strabismus, University Hospital “Sveti Duh”, Sveti Duh 64, Zagreb, Croatia; 2 Faculty of Dental Medicine and Health Osijek, Josip Juraj Strossmayer University of Osijek, Osijek, Croatia; 3 Faculty of Medicine, Josip Juraj Strossmayer University of Osijek, Osijek, Croatia; 4 University of Zagreb School of Medicine, Zagreb, Croatia; 5 Department of Ophthalmology, University Hospital Centre Zagreb, Zagreb, Croatia

**Keywords:** Macula, Angiography, Retinal vessels, Blood supply, Phacoemulsification, Cataract

## Abstract

**Background:**

The purpose of the study is to investigate the changes of macular perfusion by OCT-angiography (OCT-A) after uncomplicated phacoemulsification.

**Methods:**

OCT-A was performed before cataract surgery, 1 week, 1 month, and 3 months after surgery recording superficial vascular complex (SVC), nerve fiber layer vascular plexus (NFLVP), superficial vascular plexus (SVP), deep vascular complex (DVC), intermediate capillary plexus (ICP) and deep capillary plexus (DCP), as well as large choroidal blood vessels and choriocapillaris (CC). Explant area (EA), vessels area (VA), vessels percentage area (VPA), total number of junctions (TNJ), junctions density (JD), total vessels length (TVL), average vessels length (AVL), total number of end points (TNEP), and mean lacunarity (ML) throughout all layers were analysed.

**Results:**

Significant changes of vascular parameters in 55 eyes of 55 patients mostly reached plateau one week after surgery and remained stable up to 3 m after surgery, occurring in all retinal layers but not in choroid and CC. The greatest increase in VPA (22.79%), TVL (16.71%), AVL (166.71%) and JD (29.49%) was in SVC. On the contrary, the greatest change of ML (− 53.41%) appeared in DVC.

**Conclusions:**

This is the first OCT-A study demonstrating perfusion alterations in macula after phacoemulsification due to functional hyperaemia. We presume the effect is evoked by increased light intensity stimulation of retina after cataract removal. Accordingly, phacoemulsification in elderly population could have advantageous feature in addition to restoring visual acuity.

**Supplementary Information:**

The online version contains supplementary material available at 10.1186/s12886-021-01837-2.

## Background

The exact impact of phacoemulsification, one of the most common surgical procedures in the world [1], on macular perfusion is unknown [2, 3]. Changes in arterial blood pressure, position of blood vessels, venous return of blood and CO_2_ levels all influence eye perfusion during cataract surgery [4]. Within retina, one of the tissues with the highest metabolic requirements in the body [5, 6], three layers of the retinal blood vessels exist: superficial, deep, and intermediate layer [6]. Superficial vascular complex (SVC), formed of nerve fiber layer vascular plexus (NFLVP) and superficial vascular plexus (SVP), is located in the nerve fiber layer (NFL) and ganglion cell layer (GCL) respectively [5]. Deep vascular complex (DVC), formed of intermediate capillary plexus (ICP) and deep capillary plexus (DCP), is located deeper in the inner nuclear (INL) and outer plexiform layer (OPL) [5]. Inner plexiform layer (IPL) is thus supported by two capillary networks: SVP and ICP. The superficial layer contains arterioles, venules, and capillaries, while the deep layer consists of capillary-sized blood vessels [5].

The aim of this study was to investigate the changes of macular perfusion analysed by optical coherence tomography angiography (OCT-A) after uncomplicated phacoemulsification in normal ageing subjects. If such changes do exist, it is necessary to define whether they are favorable or adverse and thus to provide an evidence based recommendation for the indication of cataract surgery if patients can reach further benefits beyond improvement in visual acuity.

## Methods

### Patients

The aim of the study was to investigate the changes of macular perfusion by OCT-A after uncomplicated phacoemulsification. The study was conducted at the Department of Ophthalmology, University Hospital “Sveti Duh” Zagreb on one group of patients scheduled to have microincision phacoemulsification under topical anesthesia. All patients were operated by the same surgeon (BKE) and monitored and analysed by one examiner (AK). The analysed parameters refer to only one eye.

The inclusion criteria for the study were: 1. uncomplicated senile cataract; 2. cataract Pentacam® Nucleus Staging (PNS) assessed as 1, 2 or 3 by Oculus Pentacam® nucleus grading system; 3. Quality index (Q) of OCT-A images ≥30; 4. axial length (AL) 20–25 mm measured by optical biometry; 5. intraocular pressure (IOP) measured by Goldmann tonometry 10–21 mmHg; 6. systolic blood pressure (SBP) ≥ 90 and ≤ 140 mmHg, and diastolic blood pressure (DBP) ≥ 60 and ≤ 90 mmHg.

The exclusion criteria for the study were: corneal diseases, pseudoexfoliation syndrome, cataracts other than uncomplicated senile, glaucoma, age-related macular degeneration, signs of hypertensive retinopathy [7], degenerative myopia, diabetes, intraoperative and/or postoperative complications, and poor image quality.

### Methods

All patients underwent history taking, determination of best corrected visual acuity (BCVA), auto-refractokeratometry, endothelial biomicroscopy, optical biometry, arterial pressure measurement, IOP measurement by Goldmann tonometry, optical coherence tomography (OCT) of the macula, fundus examination in mydriasis and OCT-A imaging.

BCVA was determined using the ETDRS table and recorded in the logMAR unit [8]. Corneal endothelial cells were analysed by specular biomicroscope (CEM-530, Nidek, Gamagori, Japan). The AL was determined using optical biometer (IOLMaster 700®, Zeiss, Oberkochen, Germany). Arterial pressure was measured with a digital pressure gauge (M6 Comfort, Omron, Kyoto, Japan) and IOP by Goldmann applanation tonometry. Mean arterial pressure (MAP) and ocular perfusion pressure (OPP) were calculated using MAP = (SBP + 2 x DBP) / 3 and OPP = 2 x (MAP - IOP) / 3 formulas respectively. Examination of the anterior segment of the eye was performed using a slit lamp. Tropicamide 1% (Mydriacyl®, Alcon Laboratories Inc., Geneva, Switzerland) was dripped into each eye of the patient three times at intervals of 15 min to maximize mydriasis and cycloplegia. OCT (FAST mode recording 20 × 20° of the central macula using 512 A-scans × 25 sections with 240 μm distance between sections) and autofluorescence (multi-color, infrared, blue and green) were recorded using HRA + OCT Spectralis® (Heidelberg Engineering, Heidelberg, Germany). Each patient underwent objective refraction measuring using auto-refractokeratometer (Righton Speedy-K Autorefractor Keratometer, Right Group, Tokyo, Japan), imaging on Pentacam® HR device (OCULUS Optikgeräte GmbH, Wetzlar, Germany) to calculate PNS and fundus examination with an indirect ophthalmoscope.

OCT-A was performed on HRA + OCT Spectralis® device recording 10 × 10° of the central macula, accordingly 2.9 × 2.9 mm, with 512 A-scans × 512 sections, 6 μm distance between sections and resolution of 5.7 μm/pixel. Automated segmentation performed with integrated Spectralis software of superficial layer (NFLVP and SVP forming SVC) and deep layer (DCP and ICP forming DVC) of retinal vessels, CC and large choroidal blood vessels is considered reliable in case of unremarkable retinal layer pathology [9]. Recording was done using high resolution mode, reported for low measurement variability [10, 11]. In case of possible misleading artefacts, the measurement was repeated. Images with quality index Q ≥ 30, as computed by integrated software, were further analysed and carefully reviewed by two graders for accurate segmentation of the vascular layers and presence of artefacts prior to final analysis [9].

**Fig. 1:**
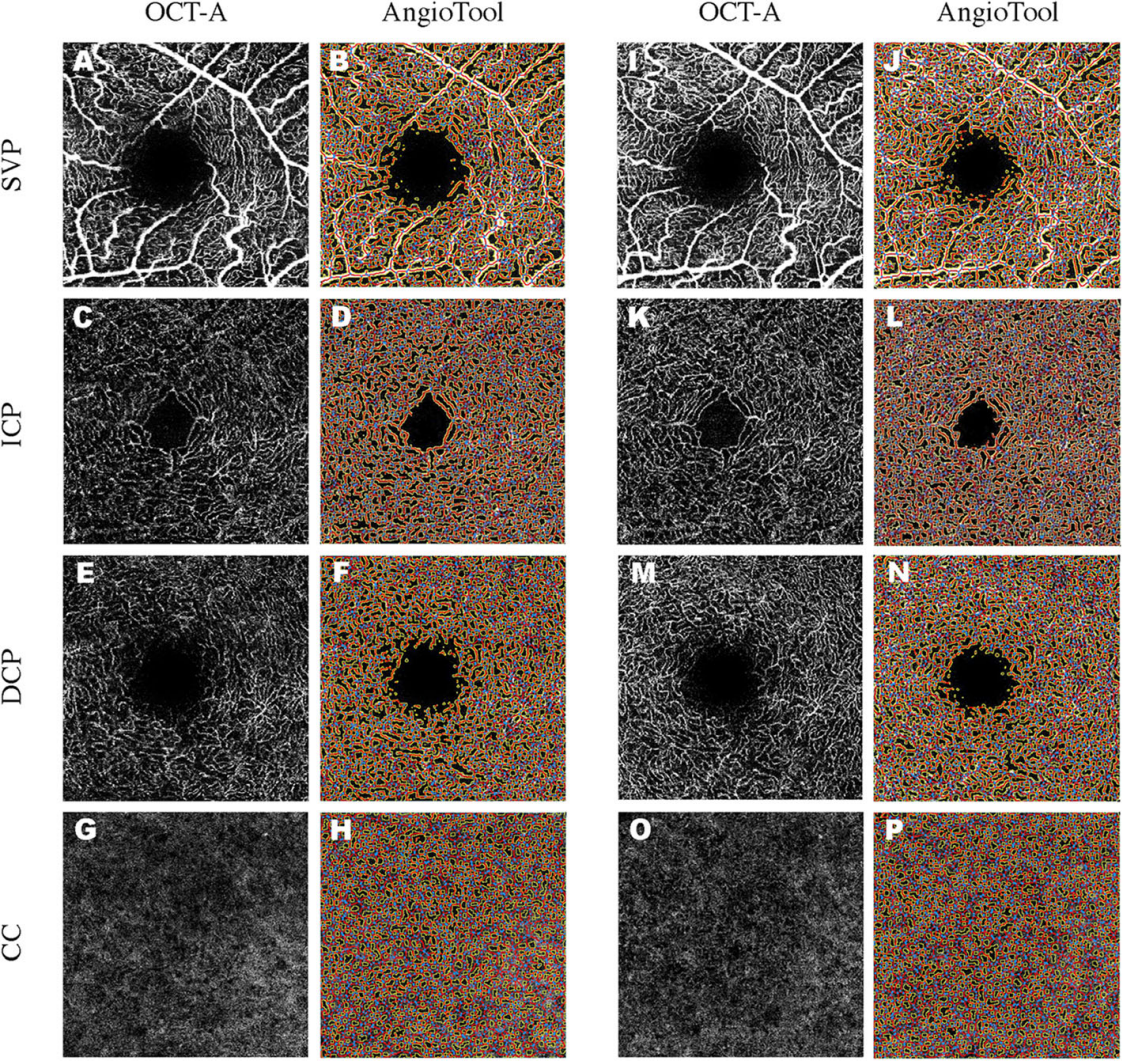
OCT-A *en-face* images (image quality Q = 35) showing binary skeletal structure in the left column **a, c, e, g, i, k, m, o** and skeletonized images with overlay of the AngioTool output in the corresponding right column **b, d, f, h, j, l, n, p** before and 1 week after surgery. The skeleton is outlined in red, vessels are presented in yellow, branching points are indicated in blue. Calculated vascular parameters are presented in the Additional file 1: Table S11

Patients were evaluated before and 7 days, 1 month, and 3 months after surgery. Images were exported to AngioTool 0.6 software for quantitative analysis [12]. A vessel is defined after segmentation as a segment between two branching points or a branching point and an end point. After segmentation, vessels were skeletonized and analysed (Fig. 1) [12]. OCT-A vascular parameters included: explant area (EA): the analysed area; vessels area (VA): area of the segmented vessels; vessels percentage area (VPA): percentage of area containing vessels inside the explant area (VA/EA); total number of junctions (TNJ): total number of vessels junctions in the image; junctions’ density (JD): number of vessel junctions per unit area (branch points/unit area); total vessels length (TVL): sum of Euclidean distances between the pixels of all the vessels in the image; average vessels length (AVL): mean length of all the vessels in the image; total number of end points (TNEP): number of open-ended segments; mean lacunarity (ML): mean lacunarity over all size boxes.

Foldable intraocular lens (Zeiss CT Lucia 611PY or AMO Tecnis PCB00) were implanted. Total cumulative dissipated energy (CDE) and total ultrasound time (PHACO time) were automatically recorded using the Centurion® Vision System (Alcon Inc., Fort Worth, USA) [13]. Postoperatively dexamethasone 0.1% drops (Maxidex®, Alcon Laboratories Inc., Geneva, Switzerland) were prescribed q.i.d. for 7 days followed by b.i.d. for 7 days.

### Statistical analysis

Statistical analysis was performed using MedCalc statistical software (MedCalc Software Ltd., Ostend, Belgium). Data are presented by median and interquartile ranges. A comparison of pre-operative values with values obtained one week, one month, and three months after surgery was made using non-parametric Friedman ANOVA test. Inter-layer comparison of vascular parameters three months after surgery was made using Student’s *t* test. The significance level was set to *P* < 0.05.

## Results

Fifty-nine patients met the inclusion criteria, yet 55 eyes of 55 patients were included in final analysis. One patient developed postoperative uveitis and was excluded from further analysis. Two patients missed the follow-up appointments. One patient developed pseudophakic macular edema (PME) 1 m after surgery based on OCT criteria [14], with no signs of PME three months after surgery. General characteristics of the participants, and surgery parameters are presented in the Additional file 1: Table S1.

### General parameters

IOP before surgery was significantly higher than one week, one month and three months after surgery. IOP one week after surgery was significantly higher than one month and three months after surgery. SBP before surgery was significantly higher than one month and three months after surgery. BCVA significantly improved one week after surgery, with further significant improvement when comparing one week and three months after surgery. DBP, MAP and OPP did not display any change (Additional file 1: Table S2).

### Quality index

Average quality of OCT-A images before and after phacoemulsification demonstrated no significant changes (Table 1).

**Table 1:** Quality index of OCT-A images before and after phacoemulsification

OCT-A image	Before	1 week after	1 month after	3 months after	*P*
Quality index	34 (31–37.5)	34 (32–36.8)	35 (32.25–37.75)	35 (33–38)	0.170

*OCT-A* optical coherence tomography angiography, *Q* quality index

This table shows median and interquartile ranges for quality index (25th and 75th percentiles)

Friedman ANOVA test, the significance level was set to *P* < 0.05

### Vascular parameters

No difference was found in EA. An increase in parameters: VA, VPA, TNJ, JD, TVL and AVL was found in all layers except in CC and choroid. A decrease in parameter TNEP was found in all layers except in choroid and NFLVP. ML decreased in all layers but choroid.

The most pronounced changes were found in NFLVP and SVC. Average vessels length has undergone the greatest change in all layers except in NFLVP. For most vascular parameters a statistically significant difference was observed between values obtained before surgery and values obtained one week, one month, and three months after surgery within which there was no statistically significant difference (Additional file 1: Table S3; Table S4).

Changes of vascular parameters in SVP, ICP, DCP and CC are presented in Fig. 1.

### Nerve fiber layer vascular plexus, superficial vascular plexus and superficial vascular complex

In NFLVP layer, VA, VPA, TNJ, JD, TVL and AVL one week after surgery were lower than one month and three months after surgery, within which there was no significant difference (Table 2). For TNEP no alterations were detected (Table 2).

**Table 2:** Statistical analysis of changes in vascular parameters in nerve fiber layer vascular plexus

NFLVP	Before	1 week after	1 month after	3 months after	*P*	Bias
EA (mm^2^)	8.3791 (8.3755–8.3804)	8.3796 (8.3770–8.3805)	8.3794 (8.3768–8.3807)	8.3794 (8.3759–8.3803)	0.740	0.05%
VA (mm^2^)	1.7746 (1.3408–2.4250)	2.3389 (1.7994–2.7072)	2.4731 (2.0283–3.0603)	2.4584 (1.9830–2.9862)	< 0.001^*^	31.72%^*^
VPA (%)	21.1911 (16.0203–28.9405)	27.9786 (21.4717–32.3195)	29.5066 (24.2454–36.5190)	29.3376 (23.6625–35.6347)	< 0.001^*^	31.67%^*^
TNJ	456 (300–725)	692 (457–871)	781 (583–1000)	729 (558–996)	< 0.001^*^	44.48%^*^
JD (junctions/mm^2^)	54.5141 (35.8141–86.5340)	82.5760 (54.5604–103.8807)	93.3164 (69.6196–119.4013)	86.9921 (66.5249–118.8970)	< 0.001^*^	44.43%^*^
TVL (mm)	66.7446 (51.4339–93.6245)	87.9231 (70.0598–102.8570)	94.7294 (76.9631–114.5119)	92.3583 (78.0480–112.1177)	< 0.001^*^	29.22%^*^
AVL (mm)	0.1761 (0.1287–0.2544)	0.2275 (0.1656–0.3089)	0.2604 (0.1823–0.3953)	0.2691 (0.1864–0.3845)	< 0.001^*^	24.72%^*^
TNEP	954 (864–1031)	984 (874–1061)	966 (855–1059)	959 (850–1124)	0.426	4.39%
ML	0.2865 (0.2111–0.4280)	0.2288 (0.1935–0.3086)	0.2311 (0.1543–0.2945)	0.2151 (0.1603–0.2506)	**< 0.001**	−31.15%

*NFLVP* nerve fiber layer vascular plexus, *EA* explant area, *VA* vessels area, *VPA* vessels percentage area, *TNJ* total number of junctions, *JD* junctions density, *TVL* total vessels length, *AVL* average vessels length, *TNEP* total number of end points, *ML* mean lacunarity

This table shows median and interquartile ranges for each parameter (25th and 75th percentile). *P* values and percentages of change are presented one week after phacoemulsification

Friedman ANOVA test, significant difference (bold values) was found for values with *P* < 0.05

*Observed values were lower one week compared to one and three months after surgery, within which there was no significant difference

In SVP and SVC an increase in VA, VPA, TNJ, JD and TVL with decrease in TNEP and ML was found one week postoperatively (Table 3; Table 4). In addition, TNEP was higher one week after surgery than three months after surgery (Table 3; Table 4). In SVP layer, TVL one week after surgery was lower than three months after surgery (Table 3). In SVC layer TNJ, JD, TVL and AVL values were lower one week compared to three months after surgery (Table 4).

**Table 3:** Statistical analysis of changes in vascular parameters in superficial vascular plexus

SVP	Before	1 week after	1 month after	3 months after	*P*	Bias
EA (mm^2^)	8.3792 (8.3777–8.3806)	8.3794 (8.3780–8.3806)	8.3801 (8.3793–8.3807)	8.3801 (8.3790–8.3809)	0.066	0.01%
VA (mm^2^)	4.4132 (3.8255–4.9965)	4.9826 (4.6831–5.2155)	5.0491 (4.7088–5.3848)	5.0094 (4.7490–5.4082)	**< 0.001**	15.00%
VPA (%)	52.6630 (45.6480–59.7126)	59.4532 (55.8881–62.2341)	60.2560 (56.1915–64.2580)	59.7772 (56.6644–64.6154)	**< 0.001**	14.93%
TNJ	1242 (1086–1377)	1398 (1337–1467)	1421 (1345–1495)	1433 (1364–1507)	**< 0.001**	16.58%
JD (junctions/mm^2^)	148.2088 (129.6466–164.2696)	166.8051 (159.6096–175.0851)	169.5579 (160.4704–178.4466)	170.9742 (162.8021–179.8361)	**< 0.001**	16.50%
TVL (mm)	135.6119 (126.7246–143.7911)	145.2219 (140.3096–149.2926)	147.1460 (142.4993–150.2033)	146.6076 (141.8954–151.0425)	< 0.001^*^	9.56%^*^
AVL (mm)	3.3768 (1.5705–5.5304)	6.7711 (4.8339–9.0807)	7.7264 (4.1957–10.0277)	7.5436 (4.5400–10.4743)	**< 0.001**	116.90%
TNEP	327 (190–447)	194 (162–235)	188 (145–243)	182 (136–220)	< 0.001^**^	−38.54%†
ML	0.04859 (0.03868–0.06534)	0.03816 (0.03027–0.04640)	0.03720 (0.02838–0.04485)	0.03701 (0.02614–0.04696)	**< 0.001**	−35.99%

*SVP* superficial vascular plexus, *EA* explant area, *VA* vessels area, *VPA* vessels percentage area, *TNJ* total number of junctions, *JD* junctions density, *TVL* total vessels length, *AVL* average vessels length, *TNEP* total number of end points, *ML* mean lacunarity. This table shows median and interquartile ranges for each parameter (25th and 75th percentile). *P* values and percentages of change are presented one week after phacoemulsification.

Friedman ANOVA test, significant difference (bold values) was found for values with *P* < 0.05

*Observed value was lower one week compared to three months after surgery

**Observed value was higher one week compared to three months after surgery

**Table 4:** Statistical analysis of changes in vascular parameters in superficial vascular complex

SVC	Before	1 week after	1 month after	3 months after	*P*	Bias
EA (mm^2^)	8.3796 (8.3770–8.3806)	8.3795 (8.3778–8.3806)	8.3801 (8.3794–8.3807)	8.3799 (8.3787–8.3807)	0.444	0.01%
VA (mm^2^)	3.5392 (2.9247–4.4603)	4.4781 (3.9746–4.7150)	4.5563 (4.0255–4.9281)	4.6398 (4.2046–5.0134)	**< 0.001**	22.82%
VPA (%)	42.2612 (34.9094–53.2560)	53.4469 (47.4008–56.2615)	54.3727 (48.0558–58.8102)	55.3807 (50.2028–59.8245)	**< 0.001**	22.79%
TNJ	1025 (794–1345)	1320 (1187–1442)	1360 (1215–1482)	1393 (1259–1501)	< 0.001^*^	29.51%^*^
JD (junctions/mm^2^)	122.3430 (94.7414–160.8583)	157.5173 (141.6555–172.0434)	162.3467 (145.0291–176.7771)	166.2187 (150.3590–179.1949)	< 0.001^*^	29.49%^*^
TVL (mm)	121.1787 (104.0833–141.6036)	140.6202 (133.4555–146.7111)	142.2354 (134.4940–148.7362)	144.0417 (136.0600–149.4173)	< 0.001^*^	16.71%^*^
AVL (mm)	1.1413 (0.6458–4.2501)	4.5943 (2.3966–6.4788)	4.5737 (2.4302–7.2035)	5.5527 (3.0329–9.1357)	< 0.001^*^	166.71%^*^
TNEP	477 (282–621)	262 (212–402)	234 (196–379)	228 (190–339)	< 0.001^**^	−39.56%^**^
ML	0.06767 (0.05250–0.1128)	0.04874 (0.03855–0.05780)	0.04805 (0.03578–0.06185)	0.04450 (0.03148–0.05804)	**< 0.001**	−44.15%

*SVC* superficial vascular complex, *EA* explant area, *VA* vessels area, *VPA* vessels percentage area, *TNJ* total number of junctions, *JD* junctions density, *TVL* total vessels length, *AVL* average vessels length, *TNEP* total number of end points, *ML* mean lacunarity

This table shows median and interquartile ranges for each parameter (25th and 75th percentile). *P* values and percentages of change are presented one week after phacoemulsification

Friedman ANOVA test, significant difference (bold values) was found for values with *P* < 0.05

*Observed values were lower one week compared to three months after surgery

**Observed value was higher one week compared to three months after surgery

### Intermediate capillary plexus, deep capillary plexus and deep vascular complex

In ICP and DVC an increase in VA, VPA, TNJ, JD, TVL and AVL along with decrease in TNEP and ML was found one week postoperatively. No difference between one week, one month and three months after surgery was observed for any parameter. In addition, the largest significant change was observed for AVL (75.78 and 113.40% respectively) (Table 5; Table 6).

**Table 5:** Statistical analysis of changes in vascular parameters in intermediate capillary plexus

ICP	Before	1 week after	1 month after	3 months after	*P*	Bias
EA (mm^2^)	8.3800 (8.3774–8.3806)	8.3797 (8.3779–8.3808)	8.3801 (8.3788–8.3809)	8.3800 (8.3786–8.3808)	0.359	0.01%
VA (mm^2^)	4.4108 (3.7545–4.8631)	4.8974 (4.6741–5.1877)	4.9264 (4.5317–5.1928)	5.0351 (4.7020–5.3053)	**< 0.001**	16.06%
VPA (%)	52.6315 (44.8284–58.0264)	58.4357 (55.8038–62.1985)	58.7958 (54.0681–61.9713)	60.0813 (56.1073–63.3031)	**< 0.001**	16.02%
TNJ	1446 (1197–1576)	1593 (1504–1675)	1603 (1452–1682)	1619 (1506–1670)	**< 0.001**	16.46%
JD (junctions/mm^2^)	172.6665 (142.9233–188.0191)	190.1043 (179.5759–200.7896)	191.3712 (173.2411–200.7275)	193.1983 (179.7861–202.8178)	**< 0.001**	16.42%
TVL (mm)	145.8206 (129.0303–153.5934)	154.3064 (150.4366–158.9588)	155.3216 (146.2016–158.3196)	155.9789 (148.7249–160.2269)	**< 0.001**	10.80%
AVL (mm)	3.1665 (1.1356–5.2864)	6.2908 (4.1408–8.3364)	6.2008 (3.8223–8.0361)	6.2737 (3.8795–8.4574)	**< 0.001**	75.78%
TNEP	366 (236–550)	233 (191–296)	236 (202–313)	231 (190–304)	**< 0.001**	−36.58%
MEL	0.03303 (0.02445–0.05412)	0.02367 (0.02006–0.02980)	0.02469 (0.01956–0.03311)	0.02373 (0.01769–0.03048)	**< 0.001**	−50.61%

*ICP* intermediate capillary plexus, *EA* explant area, *VA* vessels area, *VPA* vessels percentage area, *TNJ* total number of junctions, *JD* junctions density, *TVL* total vessels length, *AVL* average vessels length, *TNEP* total number of end points, *ML* mean lacunarity

This table shows median and interquartile ranges for each parameter (25th and 75th percentile). *P* values and percentages of change are presented one week after phacoemulsification

Friedman ANOVA test, significant difference (bold values) was found for values with *P* < 0.05

**Table 6:** Statistical analysis of changes in vascular parameters in deep vascular complex

DVC	Before	1 week after	1 month after	3 months after	*P*	Bias
EA (mm^2^)	8.3798 (8.3767–8.3807)	8.3799 (8.3788–8.3808)	8.3804 (8.3791–8.3810)	8.3799 (8.3786–8.3808)	0.128	0.02%
VA (mm^2^)	4.6295 (3.7813–5.1470)	5.2785 (5.0204–5.5748)	5.3497 (4.9653–5.6558)	5.3920 (4.9867–5.7371)	**< 0.001**	18.17%
VPA (%)	55.2483 (45.1905–61.4192)	63.0098 (59.9148–66.5252)	63.8886 (59.2817–67.4735)	64.3579 (59.5031–68.4626)	**< 0.001**	18.10%
TNJ	1601 (1270–1665)	1716 (1670–1771)	1729 (1647–1772)	1722 (1634–1780)	**< 0.001**	15.41%
JD (junctions/mm^2^)	191.0277 (151.6214–198.5983)	204.7507 (199.3202–211.4425)	206.7926 (196.6997–211.4956)	205.4722 (194.6548–212.3658)	**< 0.001**	15.34%
TVL (mm)	152.8660 (130.5779–158.5100)	160.3862 (156.7585–163.6309)	161.0969 (156.1049–163.2658)	160.5890 (155.0713–163.9713)	**< 0.001**	10.74%
AVL (mm)	3.8988 (0.9151–10.1915)	11.0566 (6.7885–23.2487)	13.4610 (7.3215–20.4125)	12.1796 (7.0645–20.5614)	**< 0.001**	113.40%
TNEP	333 (177–525)	163 (117–221)	155 (115–225)	140 (112–213)	**< 0.001**	−49.04%
ML	0.03602 (0.02654–0.07429)	0.02452 (0.01976–0.03203)	0.02530 (0.01902–0.03206)	0.02564 (0.01882–0.03265)	**< 0.001**	−53.41%

*DVC* deep vascular complex, *EA* explant area, *VA* vessels area, *VPA* vessels percentage area, *TNJ* total number of junctions, *JD* junctions density, *TVL* total vessels length, *AVL* average vessels length, *TNEP* total number of end points, *ML* mean lacunarity

This table shows median and interquartile ranges for each parameter (25th and 75th percentile). *P* values and percentages of change are presented one week after phacoemulsification

Friedman ANOVA test, significant difference (bold values) was found for values with *P* < 0.05

In DCP layer an increase in TNJ, JD, TVL and AVL and decrease in TNEP and ML was found postoperatively one week, one month and three months after surgery with no significant difference between them. VA and VPA before surgery were lower than one week, one month and three months after surgery. VA and VPA one week after surgery were lower than three months after surgery. In DCP, the largest statistically significant change in preoperative versus postoperative value was observed for AVL (103.51%) (Table 7).

**Table 7:** Statistical analysis of changes in vascular parameters in deep capillary plexus

DCP	Before	1 week after	1 month after	3 months after	*P*	Bias
EA (mm^2^)	8.3792 (8.3766–8.3803)	8.3802 (8.3790–8.3808)	8.3802 (8.3789–8.3807)	8.3801 (8.3790–8.3810)	0.357	0.01%
VA (mm^2^)	4.2319 (3.4769–4.8241)	4.9539 (4.4996–5.2425)	4.9380 (4.5294–5.3485)	4.9121 (4.5870–5.4160)	< 0.001^*^	17.65%^*^
VPA (%)	50.5055 (41.6312–57.5592)	59.1286 (53.7127–62.5724)	58.9327 (54.1117–63.8239)	58.6169 (54.7285–64.6397)	< 0.001^*^	17.59%^*^
TNJ	1375 (1078–1546)	1573 (1442–1646)	1572 (1446–1650)	1572 (1440–1654)	**< 0.001**	16.77%
JD (junctions/mm^2^)	164.1052 (128.8104–184.4084)	187.7513 (172.1331–196.4872)	187.7643 (172.532–196.884)	187.5586 (171.8801–197.3193)	**< 0.001**	16.71%
TVL (mm)	138.1963 (120.0253–150.4007)	151.3618 (144.6869–156.4053)	152.0403 (145.4315–156.7864)	151.0493 (145.3439–156.7136)	**< 0.001**	11.00%
AVL (mm)	1.9423 (0.7977–4.6726)	5.8517 (3.7833–8.4856)	5.3625 (3.2110–8.1661)	5.2044 (3.3965–8.1440)	**< 0.001**	103.51%
TNEP	411 (237–597)	239 (180–328)	219 (171–311)	225 (167–298)	**< 0.001**	−37.06%
ML	0.05597 (0.04235–0.08264)	0.04124 (0.0304–0.05118)	0.03980 (0.03073–0.04955)	0.03709 (0.03059–0.05586)	**< 0.001**	−44.00%

*DCP* deep capillary plexus, *EA* explant area, *VA* vessels area, *VPA* vessels percentage area, *TNJ* total number of junctions, *JD* junctions density, *TVL* total vessels length, *AVL* average vessels length, *TNEP* total number of end points, *ML* mean lacunarity

This table shows median and interquartile ranges for each parameter (25th and 75th percentile). *P* values and percentages of change are presented one week after phacoemulsification

Friedman ANOVA test, significant difference (bold values) was found for values with *P* < 0.05

*Observed values were lower one week than three months after surgery

### Retina

Observing the whole retina, an increase in VA, VPA, TNJ, JD, TVL and AVL with decrease in TNEP and ML was found one week postoperatively. No difference between one week, one month and three months after surgery was observed for any parameter (Table 8).

**Table 8:** Statistical analysis of changes in vascular parameters in retina

RETINA	Before	1 week after	1 month after	3 months after	*P*	Bias
EA (mm^2^)	8.3794 (8.3782–8.3804)	8.3796 (8.3781–8.3807)	8.3798 (8.3789–8.3805)	8.3800 (8.3794–8.3808)	0.567	0.00%
VA (mm^2^)	4.4237 (3.8317–4.7725)	4.8044 (4.4337–5.0568)	4.7583 (4.4779–5.1489)	4.7814 (4.4562–5.0985)	**< 0.001**	8.86%
VPA (%)	52.8002 (45.7295–56.9454)	57.3259 (52.9224–60.3462)	56.7723 (53.4809–61.4520)	57.0595 (53.1767–60.8384)	**< 0.001**	8.86%
TNJ	1544 (1302–1648)	1690 (1512–1750)	1638 (1547–1758)	1672 (1594–1747)	**< 0.001**	10.72%
JD (junctions/mm^2^)	184.2823 (155.3695–196.6816)	201.6892 (180.4112–209.4030)	195.4505 (184.6068–209.7366)	199.4898 (190.1586–208.4696)	**< 0.001**	10.71%
TVL (mm)	150.0282 (135.6528–156.1119)	158.3173 (149.9265–161.9888)	157.3335 (151.8571–162.0794)	158.2087 (153.6966–162.5753)	**< 0.001**	6.19%
AVL (mm)	2.4171 (1.0409–4.7464)	5.4592 (3.5082–7.8666)	4.8714 (3.4563–7.4278)	5.8118 (3.0205–7.7420)	**< 0.001**	87.65%
TNEP	419 (269–587)	255 (215–373)	270 (209–356)	263 (196–369)	**< 0.001**	−30.99%
ML	0.03724 (0.02724–0.05949)	0.03017 (0.02426–0.03485)	0.03040 (0.02414–0.03666)	0.02909 (0.02460–0.03849)	**< 0.001**	−30.26%

*EA* explant area, *VA* vessels area, *VPA* vessels percentage area, *TNJ* total number of junctions, *JD* junctions density, *TVL* total vessels length, *AVL* average vessels length, *TNEP* total number of end points, *ML* mean lacunarity. This table shows median and interquartile ranges for each parameter (25th and 75th percentile). *P* values and percentages of change are presented one week after phacoemulsification

Friedman ANOVA test, significant difference (bold values) was found for values with *P* < 0.05

### Choriocapillaris

In choriocapillaris TNEP was higher before surgery than one week, one month and three months after surgery, with no significant difference between them. ML before surgery was higher than one month and three months after surgery (Additional file 1: Table S5).

### Choroid

No statistically significant changes in vascular parameters were observed (Additional file 1: Table S6).

### Inter-layer differences

Morphometric differences between the vascular layers are presented online in Additional file 1: Tables S7-S10.

## Discussion

This study monitored vascular parameters of retinal blood vessels across NFLVP, SVP, SVC, ICP, DCP, DVC, as well as CC and large and medium choroidal blood vessels revealing that uncomplicated phacoemulsification significantly improved macular hemodynamics. We consider these changes to be favorable and beneficial.

### Major results

A significant increase in VA, VPA, TNJ, JD, TVL and AVL was found, followed by the decrease in TNEP and ML manifesting rise in blood supply of the central macula after phacoemulsification (Additional file 1: Table S3; Table S4). Most changes in vascular parameters were evident one week after surgery and remained stable up to three months after surgery. These changes affected all retinal layers but not CC and choroid (Additional file 1: Table S5; Table S6). Observed patterns of alterations between the retina and choroid were somewhat expected as these two layers have different hemodynamic properties.

### Functional hyperaemia

Inflammatory response after uncomplicated phacoemulsification is imputed to be the cause of increased macular thickness, reaching its maximum between one week and one month after surgery and returning to baseline after 2–6 months [2, 15, 16]. However, none of these studies actually measured the local release of inflammatory metabolites. On the contrary, the prevailing view modelled from our study results’ was that increase in macular hemodynamics could not be the result of postoperative inflammation as the changes persisted three months after the surgery, when inflammatory response should have been over [2, 15, 17–19], nor decrease in IOP due to OPP consistency (Additional file 1: Table S2) [3]. Moreover, all patients underwent uncomplicated phacoemulsification with favorable, low PHACO time and CDE (Additional file 1: Table S1), while factors contributing to neural damage and blood-brain barrier breakdown were excluded prior to enrolment.

Thus, the third mechanism, functional hyperaemia, disclosed that blood flow in retinal vasculature significantly varied due to increased intensity of light stimulus after cataract removal, in stark contrast to choroidal circulation [5]. Namely, cataract blocks up to 40% of light at different wavelengths [20]. Increase in metabolism which accompanies neuronal activity lowers O_2_ and glucose levels and leads to production of vasoactive metabolites [21]. Products of neuronal activity adenosine, lactate and arachnoidic acid cause vasodilatation and functional hyperaemia to compensate for energy consumption and increase in ganglion cell activity due to light stimulation restoring O_2_ and glucose levels [5, 22, 23]. Likewise, larger blood flow is induced after a period of dark adaptation [23], what in our case, hypothetically, we could consider cataract to be as cataracts gradually weaken the intensity of light stimuli.

### SVC vs DVC

The greatest increase in VA, VPA, TNJ, JD, TVL and AVL was found in SVC (Additional file 1: Table S4). Due to functional hyperaemia, AVL increased more than TVL suggesting coiling of blood vessels (Additional file 1: Table S4).

On the contrary, the greatest change of ML as an index for vascular structural non-uniformity, [12] appeared in DVC (Additional file 1: Table S4). These differences suggest different metabolic demands of retinal layers supplied by SVC and DVC.

NFLVP, a layer of long capillaries with small number of anastomosis located only in the posterior pole [6, 24], is presented with unique configuration (Additional file 1: Table S7) manifesting the highest number of end points and the lowest number of junctions (Table 2; Additional file 1: Table S7; Table S9).

Three months after surgery VA, VPA, TNJ, JD, TVL and AVL were significantly higher in DVC compared to SVC, while TNEP and ML were significantly higher in SVC (Additional file 1: Table S10). On the contrary, while comparing SVP and DCP (Additional file 1: Table S8), greater VA and VPA for SVP network were found likewise [10], but DCP showed higher TNJ, JD and TVL. Aforesaid attributes could correlate to the larger diameter of the SVP vasculature opposed to DVP being solely capillary meshwork [25]. Morphometry of ICP and DCP did not demonstrate significant differences (Additional file 1: Table S8).

Until this date, only two OCT-A studies analysed retinal blood vessels after uncomplicated phacoemulsification [2, 3]. Performed on a relatively small subject samples (*N* = 9 and *N* = 32), they used different OCT-A devices while Q and EA comparison before and after cataract surgery was not performed [2, 3]. Zhao et al. observed parafoveal and perifoveal blood vessels density increase one week after phacoemulsification lasting up to three months after surgery [3]. Furthermore, cataract surgery was followed by a decrease in the foveal avascular zone surface, and an increase in full and inner retinal thickness, occurring one week after surgery, and still increasing up to three months after the surgery [3]. Outer retinal thickness remained almost unchanged [3]. The vascular pattern responses observed here could give the anatomical background for the aforementioned results. Although all retinal layers demonstrated increase in perfusion, SVC underwent greater change than DVC (Additional file 1: Table S4). Inner plexiform layer contains both superficial and deep blood vessels [6]. Thus, the functional hyperaemia observed could cause an increase in full and inner retinal thickness [3]. On the contrary, we hypothesize that functional hyperaemia identified in DCP cannot elicit significant changes of outer retinal thickness as DCP is intersected between INL and OPL [6], justifying Zhao’s et al. results [3].

### Choroid

CC presented with the greatest VA, VPA, TNJ, JD, TVL and AVL, and the lowest TNEP and ML (Additional file 1: Table S5; Table S9) confirming its structure as a continuous capillary meshwork with a high number of anastomosis [25]. Opposed to the retina, we found no significant changes in the choroid (Additional file 1: Table S6) and CC (Additional file 1: Table S5), except for TNEP and ML in CC. Light stimulation has a little effect on choroidal circulation, insensitive to pO_2_ fluctuations [5]. We thus concluded that physiological requirements outlined with low PHACO time and CDE did not reach the threshold to induce outer blood-retinal barrier breakdown and inflammatory response. Further, significant decrease of TNEP and ML in CC (Additional file 1: Table S5) could result from increased demand in heat dissipation through opening of anastomoses corroborating with published studies [2, 16, 26]. Some authors hypothesized increase in subfoveal thickness to be a consequence of local choroidal inflammatory response but found no reasoning for these changes, while correlation to CDE was not reported [18, 25, 27].

### Ageing

Ageing causes altered hemodynamics and hypoperfusion mostly in neural tissues with high metabolic demand [28]. Decline in metabolic activity under physiological ageing was further supported by our study correspondingly, as retinal macular perfusion significantly increased after uncomplicated phacoemulsification.

### Image quality

One might ask if these changes in macular perfusion after surgery were the result of better image quality after cataract removal. Thus, we counterbalanced this potential bias. OCT-A may overcome early stage cataract in contrast to clinically significant ones [29]. As follows, this study included only patients with mild to moderate opacities, graded objectively (Table S1). Secondly and more important, OCT-A image quality (Q) quantified by the software integrated in HRA + OCT Spectralis® before and after surgery was statistically the same (Table 1). Consequently, perfusion changes demonstrated here were unlikely the result of the improvement of the ocular optics after cataract removal. Furthermore, different time frames of perfusion alterations were demonstrated (Tables 2-8; Additional file 1: Table S5). In addition, the latest software version of Spectralis® uses the Position Artefact Removal tool, eliminating blood movement artefacts and enabling even more distinct analysis of deeper layers [9]. With TruTrack Active Eye Tracking technology high-quality retinal imaging even with eye movements is allowed [9].

There are few limitations to this analysis. First, we reported the values for only 55 subjects of Caucasian descent for whom we did not measure retinal metabolic activity. Further studies using OCT-A are needed to establish a normative database of observed vascular parameters for other demographic variables. Second, the analysis of retinal and choroidal pathology was beyond the scope of this report.

## Conclusions

This is the first OCT-A study that has clearly demonstrated persistent increase in macular perfusion most likely due to functional hyperaemia. We presume the effect is evoked by increased light intensity stimulation of retina after cataract removal. In this study, phacoemulsification in elderly population proved as an advantageous feature in addition to restoring visual acuity. This beneficial event could facilitate the decision-making process with regard to earlier timing for cataract removal in healthy aging patients. Further studies with a longer follow-up period are warranted to validate our results and reveal temporal trends. Thus, to conclude if functional hyperaemia is a long-term condition.

## Supplementary Information

**Additional file 1: Table S1** General characteristics of patients and surgery parameters. **Table S2** Pressure parameters and visual acuity changes. **Table S3** Determined changes in vascular parameters in corresponding layers. **Table S4** Percentage of determined changes in vascular parameters in corresponding layers. **Table S5** Statistical analysis of changes in vascular parameters in choriocapillaris. **Table S6** Statistical analysis of changes in vascular parameters in choroid. **Table S7** Morphometric differences between nerve fiber layer and superficial vascular plexus, intermediate capillary plexus and deep capillary plexus three months after surgery. **Table S8** Morphometric differences between superficial vascular plexus, intermediate capillary plexus and deep capillary plexus three months after surgery. **Table S9** Morphometric differences between choriocapillaris and nerve fiber layer vascular plexus, superficial vascular plexus, intermediate capillary plexus and deep capillary plexus three months after surgery. **Table S10** Morphometric differences between deep and superficial vascular complex three months after surgery. **Table S11** OCT-A vascular parameters before and one week after phacoemulsification.

## Data Availability

The datasets generated and/or analysed during the current study are not publicly available due to extensive and large-scale datasheets but are available from the corresponding author on reasonable request.

## Abbreviations

AL: Axial length
AVL: Average vessels length
BCVA: Best corrected visual acuity
CC: Choriocapillaris
CDE: Cumulative dissipated energy
DBP: Diastolic blood pressure
DCP: Deep capillary plexus
DVC: Deep vascular complex
EA: Explant area
GCL: Ganglion cell layer
ICP: Intermediate capillary plexus
INL: Inner nuclear
IOP: Intraocular pressure
IPL: Inner plexiform layer
JD: Junctions density
MAP: Mean arterial pressure
ML: Mean lacunarity
NFL: Nerve fiber layer
NFLVP: Nerve fiber layer vascular plexus
OCT: Optical coherence tomography
OCT-A: Optical coherence tomography angiography
OPL: Outer plexiform layer
OPP: Ocular perfusion pressure
PHACO time: Total ultrasound time
PME: Pseudophakic macular oedema
PNS: Pentacam® Nucleus Staging
Q: Images quality index
SBP: Systolic blood pressure
SVC: Superficial vascular complex
SVP: Superficial vascular plexus
TNEP: Total number of end points
TNJ: Total number of junctions
TVL: Total vessels length
VA: Vessels area
VPA: Vessels percentage area
