# Supplementary material for: Macular perfusion analysed by optical coherence tomography angiography after uncomplicated phacoemulsification: benefits beyond restoring vision
Source: BMC Ophthalmol. 2021 Feb 5;21:71. doi: 10.1186/s12886-021-01837-2 (PMC7863317; doi:10.1186/s12886-021-01837-2)
Supplement: Supplementary file 1 — Additional file 1: Table S1 General characteristics of patients and surgery parameters. Table S2 Pressure parameters and visual acuity changes. Table S3 Determined changes in vascular parameters in corresponding layers. Table S4 Percentage of determined changes in vascular parameters in corresponding layers. Table S5 Statistical analysis of changes in vascular parameters in choriocapillaris. Table S6 Statistical analysis of changes in vascular parameters in choroid. Table S7 Morphometric differences between nerve fiber layer and superficial vascular plexus, intermediate capillary plexus and deep capillary plexus three months after surgery. Table S8 Morphometric differences between superficial vascular plexus, intermediate capillary plexus and deep capillary plexus three months after surgery. Table S9 Morphometric differences between choriocapillaris and nerve fiber layer vascular plexus, superficial vascular plexus, intermediate capillary plexus and deep capillary plexus three months after surgery. Table S10 Morphometric differences between deep and superficial vascular complex three months after surgery. Table S11 OCT-A vascular parameters before and one week after phacoemulsification. [file 12886_2021_1837_MOESM1_ESM.pdf]

Title: Macular Perfusion Analysed by Optical Coherence Tomography Angiography after Uncomplicated Phacoemulsification: Benefits beyond Restoring Vision

Authors: Ana Križanović, Mirjana Bjeloš, Mladen Bušić, Biljana Kuzmanović Elabjer, Benedict Rak, Nenad Vukojević

Table S1 General characteristics of patients and surgery parameters

Age (years)	70 (65-76)
Gender (male, %); (female, %)	M: 18/55, (32.7%); F: 32/55, (67.3%)
PNS (n/N)	GRADE 1: 15/55 (27.3%)<br>GRADE 2: 26/55 (47.3%)<br>GRADE 3: 14/55 (25.5%)
AL (mm)	23.54 (22.93-23.91)
CDE (%)	4.24 (3.04-5.44)
PHACO time (s)	22 (19-30)

PNS Pentacam® Nucleus Staging, *AL* axial length, *CDE* cumulative dissipated energy, *PHACO time* total ultrasound time, *n* number of patients with a certain grade, *N* overall number of patients.

This table shows distribution of gender and nuclear opalescence characteristics of patients (N = 55). Age, AL, CDE and PHACO time are presented as median and interquartile ranges (25th and 75th percentile).

Table S2 Pressure parameters and visual acuity changes

	Before	1 week after	1 month after	3 months after	*P*
IOP (mmHg)	14 (13-15)	12 (11-15)	12 (10-14)	12 (10-13)	**<0.001**
SBP (mmHg)	135 (130-140)	130 (121-140)	131 (120-128)	131 (123-139)	**0.033**
DBP (mmHg)	80 (75-84)	82 (75-86)	80 (75-89)	82 (75-87)	0.558
MAP (mmHg)	98.33 (94.00-101.50)	98.00 (92.75-103.33)	98.33 (92.08-103.33)	99.33 (92.00-102.89)	0.768
OPP (mmHg)	56.67 (52.84-58.30)	56.44 (52.33-59.28)	56.89 (53.14-60.22)	58.67 (53.56-60.17)	0.398
BCVA (logMAR)	0.32 (0.20-0.50)	0.02 (0-0.06)	0 (0-0.04)	0 (0-0.02)	**<0.001**

IOP intraocular pressure, *SBP* systolic blood pressure, *DBP* diastolic blood pressure, *MAP* mean arterial pressure, *OPP* ocular perfusion pressure, *BCVA* best corrected visual acuity.

This table shows median and interquartile ranges for each parameter (25th and 75th percentile). *P* values and percentages of change presented were obtained one week after phacoemulsification.

Friedman ANOVA test, significant difference (bold values) was found for values with $P < 0.05$.

Table S3 Determined changes in vascular parameters in corresponding layers

	EA (mm^2)	VA (mm^2)	VPA (%)	TNJ	JD (junctions/mm^2)	TVL (mm)	AVL (mm)	TNEP	ML
CC	0.104	0.107	0.069	0.742	0.755	0.218	0.334	**0.005**	**0.018**
Choroid	0.583	0.859	0.859	0.128	0.148	0.165	0.161	0.728	0.820
DVC	0.128	**<0.001**	**<0.001**	**<0.001**	**<0.001**	**<0.001**	**<0.001**	**<0.001**	**<0.001**
DCP	0.357	**<0.001**	**<0.001**	**<0.001**	**<0.001**	**<0.001**	**<0.001**	**<0.001**	**<0.001**
ICP	0.359	**<0.001**	**<0.001**	**<0.001**	**<0.001**	**<0.001**	**<0.001**	**<0.001**	**<0.001**
SVC	0.444	**<0.001**	**<0.001**	**<0.001**	**<0.001**	**<0.001**	**<0.001**	**<0.001**	**<0.001**
NFLVP	0.740	**<0.001**	**<0.001**	**<0.001**	**<0.001**	**<0.001**	**<0.001**	0.426	**<0.001**
SVP	0.066	**<0.001**	**<0.001**	**<0.001**	**<0.001**	**<0.001**	**<0.001**	**<0.001**	**<0.001**

CC choriocapillaris, *DVC* deep vascular complex, *DCP* deep capillary plexus, *ICP* intermediate capillary plexus, *SVC* superficial vascular complex, *NFLVP* nerve fiber layer vascular plexus, *SVP* superficial vascular plexus, *EA* explant area, *VA* vessels area, *VPA* vessels percentage area, *TNJ* total number of junctions, *JD* junctions density, *TVL* total vessels length, *AVL* average vessels length, *TNEP* total number of end points, *ML* mean lacunarity. The table shows *p* values determined with Friedman ANOVA test, significant difference (bold values) was found for values with $P < 0.05$.

Table S4 Percentage of determined changes in vascular parameters in corresponding layers

	EA (mm^2)	VA (mm^2)	VPA (%)	TNJ	JD (junctions/mm^2)	TVL (mm)	AVL (mm)	TNEP	ML
CC	0.00%	3.35%	3.35%	0.65%	0.64%	0.78%	21.28%	**-29.77%**	**-24.45%**
Choroid	0.01%	-1.64%	-1.65%	1.57%	1.56%	0.64%	34.91%	-4.57%	0.10%
DVC	0.02%	**18.17%**	**18.10%**	**15.41%**	**15.34%**	**10.74%**	**113.40%**	**-49.04%**	**-53.41%**
DCP	0.01%	**17.65%**	**17.59%**	**16.77%**	**16.71%**	**11.00%**	**103.51%**	**-37.06%**	**-44.04%**
ICP	0.01%	**16.06%**	**16.02%**	**16.46%**	**16.42%**	**10.80%**	**75.78%**	**-36.58%**	**-50.61%**
SVC	0.01%	**22.82%**	**22.79%**	**29.51%**	**29.49%**	**16.71%**	**166.71%**	**-39.56%**	**-44.15%**
NFLVP	0.05%	**31.72%**	**31.67%**	**44.48%**	**44.43%**	**29.22%**	**24.72%**	4.39%	**-31.15%**
SVP	0.01%	**15.00%**	**14.93%**	**16.58%**	**16.50%**	**9.56%**	**116.90%**	**-38.54%**	**-35.99%**

CC choriocapillaris, *DVC* deep vascular complex, *DCP* deep capillary plexus, *ICP* intermediate capillary plexus, *SVC* superficial vascular complex, *NFLVP* nerve fiber layer vascular plexus, *SVP* superficial vascular plexus, *EA* explant area, *VA* vessels area, *VPA* vessels percentage area, *TNJ* total number of junctions, *JD* junctions density, *TVL* total vessels length, *AVL* average vessels length, *TNEP* total number of end points, *ML* mean lacunarity.

This table shows difference between values before and after surgery calculated as a percentage of change.

Table S5 Statistical analysis of changes in vascular parameters in choriocapillaris

CC	Before	1 week after	1 moth after	3 months after	*P*	Bias
EA (mm^2)	8.3802 (8.3792-8.3808)	8.3800 (8.3791-8.3807)	8.3805 (8.3797-8.3808)	8.3806 (8.3794-8.3810)	0.104	0.00%
VA (mm^2)	6.2782 (5.9359-6.5347)	6.2919 (6.0341-6.5608)	6.3698 (6.1468-6.5764)	6.3742 (6.2331-6.5656)	0.107	3.35%
VPA (%)	74.9171 (70.8304-77.9742)	75.0905 (71.9969-78.3295)	76.0139 (73.3410-78.4711)	76.0521 (74.3739-78.3456)	0.069	3.35%
TNJ	1718 (1680-1747)	1724 (1675-1775)	1726 (1676-1773)	1722 (1655-1773)	0.742	0.65%
JD (junctions/mm^2)	205.0037 (200.4106-208.7315)	206.1781 (199.7983-211.8636)	205.9495 (200.0205-211.5691)	205.4750 (197.5789-211.5048)	0.755	0.64%
TVL (mm)	163.0203 (161.3628-164.3444)	163.4223 (161.6132-165.2547)	163.8211 (161.1174-165.4006)	163.6466 (160.6737-165.0192)	0.218	0.78%
AVL (mm)	32.9724 (18.3779-81.5003)	41.2055 (20.9563-80.7463)	41.0310 (27.3712-73.1829)	54.5891 (32.7869-82.4406)	0.334	21.28%
TNEP	105 (75-169)	88 (67-147)	84 (67-110)	84 (66-106)	**0.005**	-29.77%
ML	0.008605 (0.006593-0.01221)	0.008117 (0.006132-0.01048)	0.007327 (0.005829-0.008988)	0.006936 (0.006146-0.008365)	**0.018***	-24.45%*

CC choriocapillaris, *EA* explant area, *VA* vessels area, *VPA* vessels percentage area, *TNJ* total number of junctions, *JD* junctions density, *TVL* total vessels length, *AVL* average vessels length, *TNEP* total number of end points, *ML* mean lacunarity. This table shows median and interquartile ranges for each parameter (25th and 75th percentile). *P* values and percentages of change are presented for values one week after phacoemulsification.

Friedman ANOVA test, significant difference (bold values) was found for values with $P < 0.05$.

*Observed value before surgery was higher than one month and three months after surgery.

Table S6 Statistical analysis of changes in vascular parameters in choroid

CHOROID	Before	1 week after	1 month after	3 months after	*P*	Bias
EA (mm^2)	8.3802 (8.3791-8.3812)	8.3803 (8.3791-8.3808)	8.3799 (8.3791-8.3807)	8.3798 (8.3786-8.3809)	0.583	0.01%
VA (mm^2)	5.4907 (5.1763-5.7774)	5.4222 (5.2060-5.6690)	5.5237 (5.2998-5.7503)	5.4235 (5.2522-5.6758)	0.859	-1.64%
VPA (%)	65.5219 (61.7901-68.9811)	64.7080 (62.1141-67.6474)	65.9408 (63.2360-68.6289)	64.7279 (62.6694-67.7340)	0.859	-1.65%
TNJ	1510 (1429-1570)	1520 (1424-1586)	1528 (1451-1609)	1537 (1479-1600)	0.128	1.57%
JD (junctions/mm^2)	180.1649 (170.4431-187.2939)	181.3914 (170.2795-189.2626)	182.3895 (173.0901-191.9529)	183.3989 (176.5591-190.9233)	0.148	1.56%
TVL (mm)	151.7845 (146.4909-154.4779)	151.8237 (146.3457-155.5193)	152.3559 (148.5069-157.1590)	152.9334 (148.8406-155.3261)	0.165	0.64%
AVL (mm)	7.6142 (3.8903-12.3528)	10.3329 (5.0282-14.4431)	9.4911 (6.5935-13.0738)	9.4649 (5.2261-14.3568)	0.161	34.91%
TNEP	236 (192-317)	218 (179-295)	222 (176-268)	229 (184-294)	0.728	-4.57%
ML	0.01485 (0.01263-0.02143)	0.01510 (0.01251-0.02039)	0.01432 (0.01199-0.01802)	0.01515 (0.01232-0.01889)	0.820	0.10%

EA explant area, *VA* vessels area, *VPA* vessels percentage area, *TNJ* total number of junctions, *JD* junctions density, *TVL* total vessels length, *AVL* average vessels length, *TNEP* total number of end points, *ML* mean lacunarity..

The table shows median and interquartile ranges for each parameter (25th and 75th percentile). *P* values and percentages of change are presented one week after phacoemulsification.

Friedman ANOVA test, the significance level was set to $P < 0.05$, no significant difference was found.

Table S7 Morphometric differences between nerve fiber layer and superficial vascular plexus, intermediate capillary plexus and deep capillary plexus three months after surgery

	EA	VA	VPA	TNJ	JD	TVL	AVL	TNEP	ML
NFLVP *vs* SVP	0.154	**<0.001**	**<0.001**	**<0.001**	**<0.001**	**<0.001**	**<0.001**	**<0.001**	**<0.001**
NFLVP *vs* ICP	0.459	**<0.001**	**<0.001**	**<0.001**	**<0.001**	**<0.001**	**<0.001**	**<0.001**	**<0.001**
NFLVP *vs* DCP	0.406	**<0.001**	**<0.001**	**<0.001**	**<0.001**	**<0.001**	**<0.001**	**<0.001**	**<0.001**

NFLVP nerve fiber layer vascular plexus, *SVP* superficial vascular plexus, *ICP* intermediate capillary plexus, *DCP* deep capillary plexus, *EA* explant area, *VA* vessels area, *VPA* vessels percentage area, *TNJ* total number of junctions, *JD* junctions density, *TVL* total vessels length, *AVL* average vessels length, *TNEP* total number of end points, *ML* mean lacunarity.

Student’s t test, significant difference (bold values) was found for values with $P < 0.05$.

Table S8 Morphometric differences between superficial vascular plexus, intermediate capillary plexus and deep capillary plexus three months after surgery

	EA	VA	VPA	TNJ	JD	TVL	AVL	TNEP	ML
SVP *vs* ICP	0.172	**0.003**	**0.003**	**<0.001**	**<0.001**	**0.018**	**0.001**	**<0.001**	0.853
ICP *vs* DCP	0.235	0.069	0.069	0.859	0.859	0.869	0.550	0.072	0.445
SVP *vs* DCP	0.824	**0.035**	**0.034**	**<0.001**	**<0.001**	**<0.001**	0.155	**<0.001**	**0.009**

SVP superficial vascular plexus, *ICP* intermediate capillary plexus, *DCP* deep capillary plexus, *EA* explant area, *VA* vessels area, *VPA* vessels percentage area, *TNJ* total number of junctions, *JD* junctions density, *TVL* total vessels length, *AVL* average vessels length, *TNEP* total number of end points, *ML* mean lacunarity.

Student's t test, significant difference (bold values) was found for values with $P < 0.05$.

Table S9 Morphometric differences between choriocapillaris and nerve fiber layer vascular plexus, superficial vascular plexus, intermediate capillary plexus and deep capillary plexus three months after surgery

	EA	VA	VPA	TNJ	JD	TVL	AVL	TNEP	ML
NFLVP *vs* CC	0.596	**<0.001**	**<0.001**	**<0.001**	**<0.001**	**<0.001**	**<0.001**	**<0.001**	**<0.001**
SVP *vs* CC	0.967	**<0.001**	**<0.001**	**<0.001**	**<0.001**	**<0.001**	**<0.001**	**<0.001**	**<0.001**
ICP *vs* CC	0.203	**<0.001**	**<0.001**	**<0.001**	**<0.001**	**<0.001**	**<0.001**	**<0.001**	**<0.001**
DCP *vs* CC	0.791	**<0.001**	**<0.001**	**<0.001**	**<0.001**	**<0.001**	**<0.001**	**<0.001**	**<0.001**

NFLVP nerve fiber layer vascular plexus, *CC* choriocapillaris, *SVP* superficial vascular plexus, *ICP* intermediate capillary plexus, *DCP* deep capillary plexus, *EA* explant area, *VA* vessels area, *VPA* vessels percentage area, *TNJ* total number of junctions, *JD* junctions density, *TVL* total vessels length, *AVL* average vessels length, *TNEP* total number of end points, *ML* mean lacunarity.

Student's t test, significant difference (bold values) was found for values with $P < 0.05$.

Table S10 Morphometric differences between deep and superficial vascular complex three months after surgery

	EA	VA	VPA	TNJ	JD	TVL	AVL	TNEP	ML
DVC *vs* SVC	0.317	**<0.001**	**<0.001**	**<0.001**	**<0.001**	**<0.001**	**<0.001**	**0.001**	**<0.001**

SVC superficial vascular complex, *DVC* deep vascular complex, *EA* explant area, *VA* vessels area, *VPA* vessels percentage area, *TNJ* total number of junctions, *JD* junctions density, *TVL* total vessels length, *AVL* average vessels length, *TNEP* total number of end points, *ML* mean lacunarity.

Student's t test, significant difference (bold values) was found for values with $P < 0.05$.

Table S11 OCT-A vascular parameters before and one week after phacoemulsification

	EA (mm^2)	VA (mm^2)	VPA (%)	TNJ	JD (junctions/mm^2)	TVL (mm)	AVL (mm)	TNEP	ML
SVP before	8.3802	4.3042	51.3615	1123	134.0063	130.7938	3.2221	223	0.0638
SVP after	8.3802	4.7055	56.1502	1274	152.0250	138.5496	4.3598	208	0.0519
ICP before	8.3792	4.4997	53.7008	1417	169.1092	146.8770	2.7058	268	0.0343
ICP after	8.3813	4.9460	59.0123	1583	188.8728	154.2302	6.6762	267	0.0244
DCP before	8.3808	4.7956	57.2213	1504	179.4578	148.4679	5.4988	205	0.0531
DCP after	8.3808	5.2813	63.0197	1609	191.9864	154.0787	8.1094	166	0.0406
CC before	8.3804	6.6402	79.2349	1689	201.5471	162.3256	40.5814	64	0.0062
CC after	8.3796	6.6941	79.8856	1679	200.3676	162.3926	40.3926	53	0.0050

SVP superficial vascular plexus, *ICP* intermediate capillary plexus, *DCP* deep capillary plexus, *CC* choriocapillaris, *EA* explant area, *VA* vessels area, *VPA* vessels percentage area, *TNJ* total number of junctions, *JD* junctions density, *TVL* total vessels length, *AVL* average vessels length, *TNEP* total number of end points, *ML* mean lacunarity.
